# The results of knee manipulation for stiffness after total knee arthroplasty with or without an intra-articular steroid injection

**DOI:** 10.4103/0019-5413.41855

**Published:** 2008

**Authors:** Vineet Sharma, Aditya V Maheshwari, Panagiotis G Tsailas, Amar S Ranawat, Chitranjan S Ranawat

**Affiliations:** Ranawat Orthopedic Center, Lenox Hill Hospital, 130 East 77^th^ Street, 11^th^ Floor, New York, NY 10021, USA

**Keywords:** Arthrofibrosis, intraarticular injection, manipulation, pain control, range of motion, stiffness, total knee arthroplasty

## Abstract

**Background:**

Stiffness after total knee arthroplasty (TKA) requiring manipulation has a reported incidence of 1.3-54%. The purpose of this study was to compare the incidence of stiffness warranting manipulation using two different pain management protocols. We also studied the effect of an intra-articular injection of local anesthetic and steroid given at the time of manipulation on the range-of-motion (ROM) at last follow-up.

**Materials and Methods:**

A total of 286 TKAs (248 patients between January 2002 and December 2003) were compared to a second group of 292 TKAs (251 patients between January 2004 and March 2006). The first group received patient-controlled analgesia (PCA) for postoperative pain management. The second group had a peri-articular injection of a steroid-containing local anesthetic at the time of surgery, but no postoperative PCA. All patients undergoing manipulation in the second group also received a similar intra-articular injection at the time of manipulation as well. Only patients with minimum 12 months follow-up after manipulation were included in the study.

**Results:**

The overall incidence of stiffness requiring manipulation in both groups was similar at 2.4% and 2.1%, respectively (*P* = 0.1). The end results of manipulation with and without injection showed a significantly higher final ROM in patients who had had an injection at the time of manipulation (*P* = 0.001). The difference was due to the fact that patients who had an injection lost no motion from that achieved at the time of manipulation.

**Conclusion:**

We were unable to demonstrate a significant reduction in the incidence of stiffness after TKA using a modern pain management protocol. However, injection of a local anesthetic and steroid at the time of manipulation did have a significant influence on preserving the ROM that was obtained at the time of manipulation.

## INTRODUCTION

Limited range of motion (ROM) can compromise the restoration of function after a total knee arthroplasty (TKA), causing frustration both to the surgeon and the patient. Although the incidence of stiffness after TKA requiring a manipulation has been reported to be as high as 54%,^1^,^2^ most contemporary studies report it as 1.3 -13.5%.^3^–^12^ The etiology of post-TKA knee stiffness is multifactorial and postoperative pain control is an important determinant.^6^,^7^,^9^,^13^,^14^

Perhaps the most significant advancement in hip and knee replacement in this decade has been the continuous evolution of the pain management protocol.^15^ Since January 2004, we have been following a multimodal approach, which emphasizes patient education, preemptive analgesia, use of peri-articular injections and avoidance of parenteral narcotics, especially patient-controlled analgesia (PCA).^16^ Better postoperative pain control had led to accelerated rehabilitation with increased patient satisfaction and earlier return to function.^16^ These encouraging results with the new protocol prompted us to ask whether the incidence of manipulation after TKA would also decrease with improved postoperative pain control. We also sought the influence of the repeat intra-articular injection at the time of the manipulation on the final range of motion.

## MATERIALS AND METHODS

This was a retrospective study of two consecutive primary TKA groups with different pain management protocols. Between January 2002 and December 2003, 286 primary TKAs (248 patients) were performed at our institution by the senior surgeon (CSR). These included 38 bilateral TKAs, 24 being single-stage. All patients were implanted with posterior stabilized (PS) designs, which consisted of 204 mobile-bearing P.F.C.^®^ Sigma™ RP; 48 fixed-bearing P.F.C.^®^ Sigma™ and 34 all-polyethylene Knee systems (DePuy Orthopedics, Inc., Johnson and Johnson, Warsaw, IN). No local periarticular injection was given at the time of surgery for pain control. The details of the rehabilitation protocol used have been described earlier by the authors.^17^ The postoperative pain management consisted of continuous postoperative epidural anesthesia for 24-48 h in conjunction with adjuvant femoral nerve block for 24 h, with supplemental PCA and oral narcotics as necessary. Continuous passive motion (CPM) for assisted ROM was started on postoperative Day 1 from 0-60° and increased as tolerated. Closed suction drain was removed after 24 h and compression dressing was removed after 48 h. Mechanical compression boots were used continuously for 72 h. Physical therapy was started on postoperative Day 1 to aid in progressive, protected ambulation as well as isometric and ROM exercises. Warfarin was used for deep vein thrombosis prophylaxis with a target International Normalized Ratio (INR) of 1.5-2. A routine Doppler scan was done on postoperative Day 2. If the scan was negative, patients were started on aspirin; otherwise warfarin was continued for six weeks with regular INR monitoring. High-risk patients were continued on warfarin for six weeks anyway. Most patients were preferably discharged to home, or to inpatient rehabilitation, or to a skilled nursing home facility by postoperative Day 3 in consultation with social worker and home health services.

The second group consisted of 292 primary TKAs (251 patients) performed between January 2004 to March 2006 by two surgeons (CSR and ASR). These included 41 bilateral procedures, 28 being single-stage. The designs used in this group were also PS and included 149 fixed-bearing P.F.C.^®^ Sigma™, 38 mobile-bearing P.F.C.^®^ Sigma™ RP and 105 mobile-bearing high-flexion P.F.C.^®^ Sigma™ RP-F Knee systems (DePuy Orthopedics, Inc., Johnson and Johnson, Warsaw, IN). These patients received a multimodal pain management protocol, including a peri-articular injection of a steroid-containing local anesthetic at the time of surgery [Tables 1–2].^15^,^16^ The multimodal protocol uses the principles of preemptive analgesia and avoids parenteral narcotics.^15^,^16^ No postoperative epidural or regional blocks was used in this group. By avoiding the use of parenteral narcotics and regional blocks, most side-effects like respiratory depression, ileus, hypotension, bradycardia, pruritis, urinary retention, cognitive effects, nausea and vomiting were also minimized. Apart from the difference in pain management protocol, the rehabilitation protocol was essentially similar to the first group.

**Table 1:** Ranawat orthopedic center (ROC) cocktail^11^

Medication	Strength/dose	Amount
*First injection*		
Bupivacaine	0.5% (200-400 mg)	24 cc
Morphine sulphate	8 mg	0.8 cc
Epinephrine (1:1000)	300 μg	0.3 cc
Methylprednisolone	40 mg	1 cc
acetate		
Cefuroxime	750 mg	10cc (reconstituted in normal saline)
Sodium chloride	0.9%	22 cc
*Second injection*		
Bupivacaine	0.5%	20 cc
Sodium chloride	0.9%	20 cc

Clonidine transdermal patch applied in operating room (100 μg/24 h) No steroid in diabetics, immunocompromised, elderly (>80 years) or revisions Use vancomycin if allergic to penicillin

Patients in both groups were discharged after they had attained the goals of walking independently with support for at least 50 feet, transferring independently in and out of bed and toilet, and having at least 70-80° of flexion. Mostly, this was attained by the third postoperative day. All patients had access to physiotherapy after discharge.

All patients were evaluated at six weeks after surgery. Knee society scores (KSS)^18^ and ROM (with a goniometer) were recorded at each visit. Any patient with less than 90° of knee flexion, more than 15° of flexion contracture or an arc of motion of less than 70-80° was given an additional two to three weeks of extensive and supervised physical therapy aimed at increasing ROM. These patients were again re-evaluated after two to three weeks and those not improving were considered for manipulation. A proper evaluation to look for any other possible cause of stiffness like implant malposition, patello-femoral joint overstuffing, patella baja, patellar maltracking, or infection was done prior to manipulation. Preoperative ROM and the ROM attained at the time of surgery against gravity after capsular closure was taken into consideration while deciding about the need for manipulation.^2^,^19^–^22^

The technique of manipulation was the same for all patients. After induction of short general anesthesia, the hip was flexed to 90° and the knee was gently manipulated into flexion (by grasping the proximal part of the leg rather than distal) until the breaking of adhesions was felt and often heard and when a firm end point was reached. The ROM attained at the time of manipulation was recorded. In the second group of patients, an injection containing morphine, methylprednisolone acetate and bupivacaine [Table 1] was injected under aseptic conditions after manipulation. All patients were discharged home the day of manipulation with adequate pain control and cryotherapy. Physiotherapy, including CPM was started the next day. All patients were followed up at two weeks, six weeks, three months and then yearly thereafter if they were progressing as expected. Institutional board review approval was obtained for this study.

**Table 2:** Sites for intraoperative injection during TKA

First injection (before final implantation)
Posterior capsule Posteromedial and posterolateral soft tissue
Second injection (after final reduction)
Extensor mechanism Synovium Capsule Pes anserinus, antero-medical capsule and periosteum llio-tibial band Collateral ligaments

## Data analysis

We looked for the difference in incidence of manipulation between the two groups as well as the difference between the two groups as regards to gain in ROM before and after manipulation. The loss of motion, if any, between manipulation and final follow-up was also compared. All data was analyzed using a SPSS statistical package (SPSS, Chicago, IL). Continuous variables were analyzed using the student's t-test and categorical values were analyzed using the chi-square test. Mann-Whitney U was used for nonparametric values. Correlation was obtained with the degree of deformity and the rate of manipulation. A *P* value of <0.05 was considered statistically significant.

## RESULTS

The demographic for both the groups as a whole was typical for a TKA population, with no significant difference between the two groups (*P* = 0.2). The preoperative diagnosis was mostly osteoarthritis. The data on the patients requiring manipulation is shown in [Table 3].

Of the 286 TKAs done between January 2002 and December 2003 (Group 1), the incidence of stiffness requiring manipulation was 2.4% (seven knees in seven patients). The mean time for manipulation from the index procedure was 10.5 weeks (6-16). Four knees had a varus alignment (mean 8°, range 4-15°) before surgery and three had a valgus alignment (mean 7°, range 4-10°). One patient had prior knee arthroscopy. Radiographic review of these patients before manipulation did not show any evidence of mal-alignment. One of these seven knees developed a postoperative patella baja. One patient failed to achieve adequate ROM even after manipulation and underwent a complete revision.

Of the 292 primary TKAs done between January 2004 and March 2006 (Group 2), the incidence of manipulation was 2.1% (six knees in five patients) with no statistical difference between the two groups (*P* = 0.1). The mean time for manipulation from the index procedure was 9.6 weeks (7-13). Four knees had varus alignment (mean 7°, range 2-17°) before surgery and two had a valgus alignment (mean 5°, range 3-7°). No patient had prior knee surgery. None of these patients had radiographic evidence of malalignment or postoperative patella baja. There was no re-operation in this group of patients. No patient had any contraindication to steroids.

The mean gain in ROM at the time of manipulation was 40° (20-50°) in Group 1 as compared to 33° (25-45°) in Group 2. The gain in both groups was significant (*P* = 0.001) but there was no statistical difference between the two groups (*P* = 0.1) However, the mean gain in ROM at final follow-up was only 28° (15-38°) in Group 1 with a mean loss of 12° (5-22°) from the time of manipulation. In contrast none of the patients in Group 2 lost any motion (*P* = 0.001). There was no correlation between the degree of deformity and the incidence of manipulation (r = 0.1, *P* = 0.7). Although there was no difference in manipulation rates for different implants, the high flexion type had no incidence of manipulation.

## Complications

There were no complications in either group at the time of manipulation. One patient in the group with no intra-articular injection failed to gain adequate motion and underwent a complete revision. The ROM in that patient at the final follow up was 5-100°.

## DISCUSSION

While the primary aims of TKA are pain relief and restoration of mobility, an adequate ROM is also desirable. About 67° of knee flexion is required during swing phase of the gait, 83° to ascend stairs, 90-100° to descend stairs, 93° to rise from a standard chair, and up to 105° to rise from a low chair or tie a shoelace.^23^ Some populations, especially in Asian countries, may need flexion in the range of 125-135° for personal hygiene, feeding and religious purposes.

**Table 3:** The data on the patients requiring manipulation

Parameters	First group (without intra-articular injection)	Second group (with intra-articular injection)
Incidence of manipulation	7 (2.4%)	6 (2.1%)
Age (years)	65 (54-80)	65 (54-72)
Gender (M:F)	5:2	1:5*
Mean time for manipulation (weeks)	10.5 (6-16)	9.6 (7-13)
Mean follow-up (months)	20.6 (12-38)	14.6 (12-18)*
Mean preoperative ROM	101° (70-120)	86° (30-125)
Mean preoperative KSS (knee)	53 (43-75)	53 (43-60)
Mean preoperative KSS (function)	55 (45-70)	49 (40-60)
Mean postoperative ROM	64° (40-85)	77° (70-85)
ROM at manipulation	104° (60-120)	110° (105-130)
Mean ROM at last follow-up	92° (40-120)	111° (105-131)*
Mean KSS (knee) at last follow-up	83 (56-95)	88 (73-97)
Mean KSS (function) at last follow-up	85 (45-100)	90 (80-100)

* *p* <0.005, KSS - Knee Society Scores^18^ ROM - Range of Motion

The etiology of post-TKA stiffness is multi-factorial and can be divided into patient factors, surgical technique and postoperative factors.^2^–^14^,^19^–^27^ Decreased preoperative ROM has been shown to be one of the most important determinants of final ROM.^19^–^21^ Patients with osteoarthritis, post-traumatic arthritis and previous surgery are more prone to develop stiffness. A wide variety of surgical factors can predispose to stiffness such as using a poor implant design, over-sizing the femoral component, over-stuffing the patello-femoral joint, elevating the joint line, failing to balance the knee and the intraoperative ROM gained after capsular closure. Postoperative rehabilitation, patient motivation, biological predisposition to arthrofibrosis, infection, heterotrophic ossification, patellar complications and the individual's response to pain are other factors which can lead to the development of stiffness. Interestingly, Mauerhan *et al.*, showed that the manipulation rates have risen with decreasing hospital stay over the years.^12^ They attributed it to less exposure to physiotherapy. Gender has been shown to influence the final ROM (less in females).^2^,^5^,^19^ Although, there were more females in Group 2 needing manipulation, we do not think this to be clinically significant with the numbers available.

The management of stiffness after TKA depends on the time elapsed since surgery as well as the identification of potential factors which predispose to stiffness. Since pain is an important determinant of postoperative stiffness, identification of 'painful TKA' (15% of all TKAs) is essential during the first postoperative visit and guidelines have been recommended by the senior author.^17^ If significant pain (more than 3 out of 10 on a visual analog pain scale or pain requiring regular narcotics) with associated limitation of function persists after uncomplicated TKA, further workup is mandatory. First, infection and mechanical instability must be ruled out. The patient is then begun on a prolonged pain management protocol. Progress is monitored closely, with continued intensive supervised physical therapy and regular follow-ups. In refractory cases in which no surgical intervention is deemed appropriate, patients are referred to a pain management consultation.

The treatment options of stiffness following TKA include manipulation, arthroscopic or open debridement and revision surgery.^2^–^14^ Although late manipulation has also shown to improve ROM, it appears to have notably better results when undertaken within the first three months after TKA.^2^,^3^,^5^–^9^,^11^,^13^ The gain in ROM achieved at the time of anesthesia may not be maintained over time.^2^,^4^,^7^,^8^ This is partially attributed to pain which most patients experience with stiff knees. Moreover, manipulations may be associated with surgical risks, such as anesthetic complications, rupture of extensor mechanism, supracondylar fracture, wound dehiscence, hemarthrosis, myositis and even death due to fatal pulmonary embolism, and thus the decision for manipulation needs to be carefully undertaken.^2^,^4^,^5^,^9^,^12^,^14^

The purpose of using an intra-articular injection was to provide better post-manipulation pain control as well as reduce the overall inflammatory response and subsequent scar formation. We are aware of only two reports where an intra-articular injection had been used during manipulation^6^,^7^. Maloney^6^ used 20 ml of 0.25% bupivacaine with epinephrine just after induction of anesthesia and then manipulated the knees. The mean ROM achieved was 111° and this was maintained at one year. Scranton^7^ used a similar 6 ml injection with addition of 40 mg (1 ml) of methyl prednisolone acetate. The mean ROM achieved was 108° and 98° in the patients manipulated before or after 12 weeks of the TKA respectively. However, no final follow-up was presented. In another study, the use of prolonged epidural analgesia after manipulation has shown to increase the mean ROM from 71° to 102° at a mean of 18.4 months with successful results in 47% of cases.^28^ The drawback of these reports is that there was no control group for meaningful comparison of benefit of the local or epidural injections.

The overall incidence of manipulation in our series (2.1%) is favourable to most contemporary reports in the literature. But this incidence was not statistically different whether or not a peri-articular injection of cocktail was given at the time of TKA. This may be due to the high threshold the senior author has for considering manipulation. This may also suggest that adequate pain control may be even more important after manipulation to maintain the gained ROM.^6^,^7^,^28^ We have previously shown that an injection of these medications at the time of TKA helps in controlling postoperative pain and in achieving early functional milestones.^16^ However, in this study, it had a lesser influence on the incidence of stiffness or the need for manipulation. This confirms the fact that the cause of stiffness after TKA is multi-factorial. Our study does confirm that that manipulation (even beyond three months) after TKA does help in gaining ROM. Patients in both groups had a significant gain in motion after manipulation. However, the most significant finding of this study was that the patients with an injection along with manipulation were able to maintain their gained motion at last follow-up.

The retrospective nature of this study adds to its limitation. Although significant differences could be seen between the two groups, the low incidence of manipulation in each group may not provide adequate power to this study. A large prospective randomized multicentric trial may be able to provide more insight. However, the fact that no patient in the second group lost any motion gained during manipulation does point towards the advantages of the intra-articular injection. The other differentiating variable in this study could be the use of a high-flexion design in the second group (with no manipulation). However, recent Level 1 studies have shown no significant difference in maximal knee flexion in patients receiving a standard or a high-flexion knee.^29^,^30^ Although the follow-up was shorter in Group 2, it has been shown that evaluation at three to six months correlates well with the final outcome.^4^,^14^

## CONCLUSION

Based on our present study, we recommend that manipulation should be considered in patients after TKA if they have not achieved an adequate ROM. We were unable to demonstrate a significant reduction in the incidence of stiffness after TKA using a modern pain management protocol. However, injection of a local anesthetic and steroid at the time of manipulation did have a significant influence on preserving the ROM gained during manipulation.
